# Mechanism of tau transfer: insights from cell culture and animal models

**DOI:** 10.1186/1750-1326-8-S1-P50

**Published:** 2013-09-13

**Authors:** Jessica Wu, Dara Dickstein, Karen Duff

**Affiliations:** 1 Taub Institute for Alzheimer’s Disease Research, Columbia University, New York, NY, USA; 2 Fishberg Department of Neuroscience, Icahn School of Medicine at Mount Sinai, New York, NY, USA

## Objective

Tau inclusions composed of tau fibrils are one of the key hallmarks of Alzheimer’s disease and other tauopathies. Studies from transgenic animal models and human AD pathological cases suggest that tau inclusions spread from the transentorhinal cortex to mono and transynaptically connected regions of the hippocampus and neocortical regions. This study examines cell-to-cell propagation of pathological tau in cell culture and in vivo animal models.

## Methods

To study transfer between donor and recipient cells, donor primary neurons isolated from human tau expressing mice (line rTg4510) were co-cultured in microfluidic(MF) chambers with recipients that were either GFP expressing tau knockout, or wildtype neurons (model one). In another model (model two) in which more aggregates were formed, fluorescently tagged pro-aggregating domains of tau (RD) were expressed in neurons using lentivirus; mCherry was used to label the recipient cell population. Immunofluorescence (IF) and live imaging were performed to examine transfer of tau between donor and recipient neurons. To further increase tau aggregate formation (model three), brain-derived tau filaments or full length recombinant tau were used as seeds to induce endogenous tau templating, and the effect on propagation was monitored. To gain insight into possible mechanism of tau transfer between entorhinal cortex (EC) and mono-synaptically connected hippocampal cells, immuno-electron microscopy (IEM) was used to examine tau distribution in donor and recipient cells.

## Results

1. In both models one and two, endogenously produced tau transferred from donor to recipient cells. Live imaging and IF revealed that donor tau was localized, and accumulated into synapses derived from recipient cells.

2. In model three, exogenous aggregated tau was taken up and induced templating of endogenous full-length tau or RD. These tau aggregates transferred from donor to recipient cells in MFs.

3. Tissue IEM showed human tau in pre-synaptic terminals derived from EC neurons that express tau transgene, and in post-synaptic compartments derived from recipient granule cell dendrites in the hippocampus.

## Conclusions

Our data demonstrates that endogenous produced, physiologically relevant tau, aggregates derived from tau fragments and tau generated by templating transferred between neurons in culture. Current data implicates synapses as a point of accumulation for tau during propagation *in vitro* and *in vivo* models. Future studies will elucidate the relevance of synaptic accumulation and the mechanism by which propagation occurs.

